# City-scale GPS data reveals impact of spatial configuration and dedicated infrastructure on e-scooter route choice

**DOI:** 10.1038/s41598-025-06938-2

**Published:** 2025-07-07

**Authors:** Hans-Heinrich Schumann, He Haitao, Adrian Meister, Asya Natapov, Mohammed Quddus

**Affiliations:** 1 School of Architecture, Building and Civil Engineering, Loughborough University, Loughborough, UK; 2 Institute for Transport Planning and Systems (IVT), ETH Zurich, Zurich, Switzerland; 3 Centre for Transport Engineering and Modelling, Department of Civil and Environmental Engineering, Imperial College London, London, UK

**Keywords:** Civil engineering, Psychology and behaviour

## Abstract

Shared e-scooter use has rapidly expanded in major cities worldwide, offering promising solutions for sustainable transport and new data sources to advance the science of cities. This study leverages a city-scale GPS dataset of 14,029 e-scooter trips recorded over a three-month period in 2021 within the Mannheim/Ludwigshafen metropolitan area in Germany. For the first time, our analysis integrates the discrete choice modelling framework with space syntax theory using such large-scale revealed preference data, uncovering new insights into the impact of spatial configuration on routing behaviour. The results highlight the significant role of spatial configuration in e-scooter routing, with space syntax metrics consistently improving model performance and suggesting that riders avoid both places that are not well-integrated on a regional and highly accessible on a local level. Results also reveal that dedicated bicycle infrastructure, including bike lanes and tracks, reduces perceived travel distance by over 51% for e-scooter riders. Additionally, riders exhibit context-dependent behaviour, favouring pedestrian spaces during busy weekdays while avoiding them at other times. These insights can guide policymakers in designing micro-mobility-friendly urban environments.

## Introduction

E-scooters have become an integral part of urban transport systems in cities worldwide. Their electric power, silent operation, speed without requiring muscle effort, compact size, and flexibility make them a viable option for safe^1^, efficient^2,3^, environmentally friendly^4,5^, and equitable^6,7^ mobility. However, they also pose significant challenges concerning safety^8^, cluttering^9^, liability^10^, and appropriate traffic rules^11^, leading to the recent banning of shared e-scooters in major cities like Madrid, Melbourne, and Paris^12–14^. This controversy underscores the urgent need for better understanding and management of this emerging mode of transport^15,16^.

Meanwhile, e-scooters are a rich source of passively generated big data^17,18^, offering new opportunities to advance the science of cities such as space syntax theory, which aims to understand the relationship between the urban environment and people’s behaviour^19^. Rooted in graph theory-based representation, space syntax provides analytical methods to assess spatial configuration^20,21^, which refers to the overall structure of urban spaces^22^. This sociospatial theory^23^ has been used to explain a plethora of social phenomena, ranging from cultural influences on architecture^24^ to social exclusion^25^, crime patterns^26^, and economic activity^27^.

The relationship between spatial configuration and human movements has been a key research focus. The theory of natural movement –an extension of space syntax theory—posits that the configuration of space is the primary factor in explaining movement patterns^28^. Studies based on the theory of natural movement have investigated the influence of spatial configuration on the flow of pedestrians^23,28–34^, cyclists^30,35,36^, and cars^37^. However, in addition to not having examined the flow of e-scooters, they rely heavily on manual counting, a labour-intensive and error-prone method unsuitable for large-scale analysis and unable to capture temporal variations. Furthermore, they mainly cover accumulated flows without considering individuals’ traces, thus falling short of being able to explain the travellers’ decision-making processes and resulting in a lack of intuitive policy recommendations.

As a result, space syntax has seen limited integration into planning practice^38^, despite the fact that understanding routing behaviour is fundamental to state-of-the-art transport modelling approaches, such as the four-step algorithm^39^ and agent-based modelling^40^, which are crucial to assess and quantify the impacts of demographic^41^, land use^42^, and infrastructure changes^43^ on transport system performance. While route choice models have been extensively studied for traditional transport modes such as driving^44–47^, walking^48–50^, cycling^51,52^, and public transport^53,54^, route choice modelling for e-scooters remains in its infancy, so far limited to small-scale, controlled studies^55^ and with restricted geographical and demographic scope^56–58^. Filling this critical gap can help cities integrate e-scooters more effectively into their transport systems, maximising their benefits while addressing associated challenges.

This study addresses this research gap by developing the first e-scooter route choice model at a city scale, leveraging vehicle tracking data from 14,029 trips recorded from June to August 2021 in the Mannheim/Ludwigshafen metropolitan area in Germany. By analysing these trips, we uncover how various factors influence the likelihood of individuals selecting specific paths, including for the first time understanding the impact of spatial configurations on routing behaviour. The methodology involves generating a choice set for each observed trip, comprising alternative routes, and applying a discrete choice modelling framework to compare these with the actual routes taken. Furthermore, by estimating models for different time periods, we are able to identify temporal variations in e-scooter users’ route choices for the first time. Attributes tested include route length^51,57,59^, the availability of dedicated cycling infrastructure to mitigate conflicts with motorists^52,60–64^, the frequency and angles of turns^30,31,61–63,65^, and spatial configuration (Table 1).

**Table 1:** Methodology structure.

	Model 1	Model 2	Model 3	Model 4
***Attributes***				
Infrastructure type	✓			✓
Route length	✓			✓
Turns		✓		✓
Spatial configuration			✓	✓
***Time periods***				
Weekday low	✓	✓	✓	✓
Weekday high	✓	✓	✓	✓
Weekend low	✓	✓	✓	✓
Weekend high	✓	✓	✓	✓

By leveraging passively generated big data^17^ on a city scale, this study offers new insights into e-scooter routing behaviour at a high spatio-temporal resolution. These insights are valuable for both research and practice, as they enhance our understanding of the applicability of space syntax theory while supporting urban planning with a scalable, data-driven approach to inform policy and infrastructure development^38^. Moreover, this study advances space syntax theory by integrating spatial configuration parameters into discrete route choice models, revealing how spatial configuration influence the routing behaviour.

## Results

The nomenclature used in the results and the subsequent methods section is presented in Table 2.

**Table 2:** Nomenclature.

Symbol	Description
*AI(u)*	Angular Integration of segment u
*β*_*Cycle lane*_	Cycle lane parameter of route choice model
*β*_*Cycle track*_	Cycle track parameter of route choice model
*β*_*jk*_	*k*-th parameter of alternative *j*
${\beta }_{j}^{PS}$	Path Size parameter of alternative *j*
*β*_*k*_	*k*-th parameter of route choice model
*β*_*length*_	Length parameter of route choice model
*C*	Choice set
*CH*_*angular*_*(u)*	Angular Choice of segment *u*
*γ*_*i,i*+*1*_	Angle between *i*-th and *i* + *1*-th segment within a path
*d*_*angular*_*(u,v)*	Angular distance between segments *u* and *v*
*d*_*walking*_*(u,v)*	Walking distance between segments *u* and *v*
*δ*_*ij*_	Binary variable of segment *i* within route *j*
*δ*_*t*_	Binary variable of segment *t*
*f*	Index of a segment
*g*	Index of a segment
*h*	Index of an hour of the week
*i*	Index of a segment of a path *P*
*j*	Index of an alternative route
*k*	Index of a route attribute
*L*_*j*_	Path length of alternative *j*
*l*_*i*_	Segment length of segment *i*
*N*	Number of segments within a network
*N*_*P*_	Number of segments within path *P*
*n*_*ave,h*_	Average trips at hour *h* of the week
*n*_*ave,total*_	Average hourly trips over study period
*NACH(u)*	Normalised Angular Choice of segment *u*
*NAIN(u)*	Normalised Angular Integration of segment *u*
*P*	Index of a path
*P*_*j*_	Choice probability of alternative *j*
${\mathcal{P}}_{u,v}$	Set of paths between segments *u* and *v*
${\mathcal{P}*}_{f,g}$	Set of shortest paths between segments *f* and *g*
*PS*_*j*_	Path Size attribute of alternative *j*
*s*	Standard deviation of average hourly trips
*t*	Index of routes within ${\mathcal{P}*}_{f,g}$
*TD(u)*	Total Depth of segment *u*
${\Gamma }_{j}$	Set of segments in route *j*
*u*	Index of a network segment
*V*_*j*_	Systematic utility of alternative *j*
*v*	Index of a network segment
*VoD*	Value-of-distance
*w(γ*_*i,i*+*1*_*)*	Weight of angle *γ*_*i,i*+*1*_
*X*_*jk*_	*k*-th attribute of alternative *j*
*Z*_*h*_	Deviation of average e-scooter traffic at hour *h* from weekly mean

### Spatio-temporal data overview

There are, on average, 2.6 e-scooter trips per hour over the study period. As shown in Table 3, this number varies by hour of the day and day of the week, with the average peak of e-scooter trips reached on Saturdays between 8 and 9 pm, and the average minimum is observed on Wednesdays between 4 and 5am. Across the days of the week, more e-scooter trips tend to take place in the afternoon than in the morning times, with Sunday through Wednesday having a below-average number of e-scooter trips while the number of e-scooter trips on Thursday to Saturdays is above average. Average Mondays, Tuesdays, and Thursdays have a morning peak, though on a limited scale with below-average trip numbers, between 8 and 9am, and all days of the week display two peak hours in the second half of the day, one in the afternoon, and the second after 7 pm.

**Table 3:**
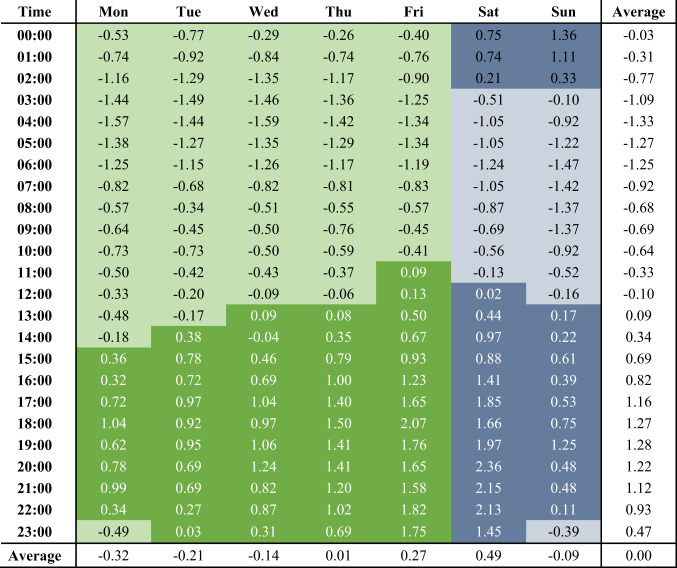
Variations of average trips per hour by day of the week, expressed in standard deviations Z_h_ with n_ave,total_ = 2.63 and s = 3.02. Hourly classification as , , , .

To facilitate the analysis of route choices of e-scooter users and identify potential temporal variations, the analysed trips are classified based on whether they are made during a time of below-average or above-average e-scooter traffic and whether the trip started during a weekday or on the weekend. In summary, four time periods are considered: (a) weekday low traffic, (b) weekday high traffic, (c) weekend low traffic, and (d) weekend high traffic. The temporal distribution of these time periods over the hours of the week is displayed with varying font colours in Table 3. For the reader’s information, Table 3 also includes the deviation *Z*_*h*_ of each hour *h* of the week’s mean trip number *n*_*av*e*,h*_ from the average number of trips per hour *n*_*ave,total*_ expressed in standard deviations *s*, so that *Z*_*h*_ = (*n*_*av*e*,h*_—*n*_*ave,total*_)/*s*.

**Fig. 1:**
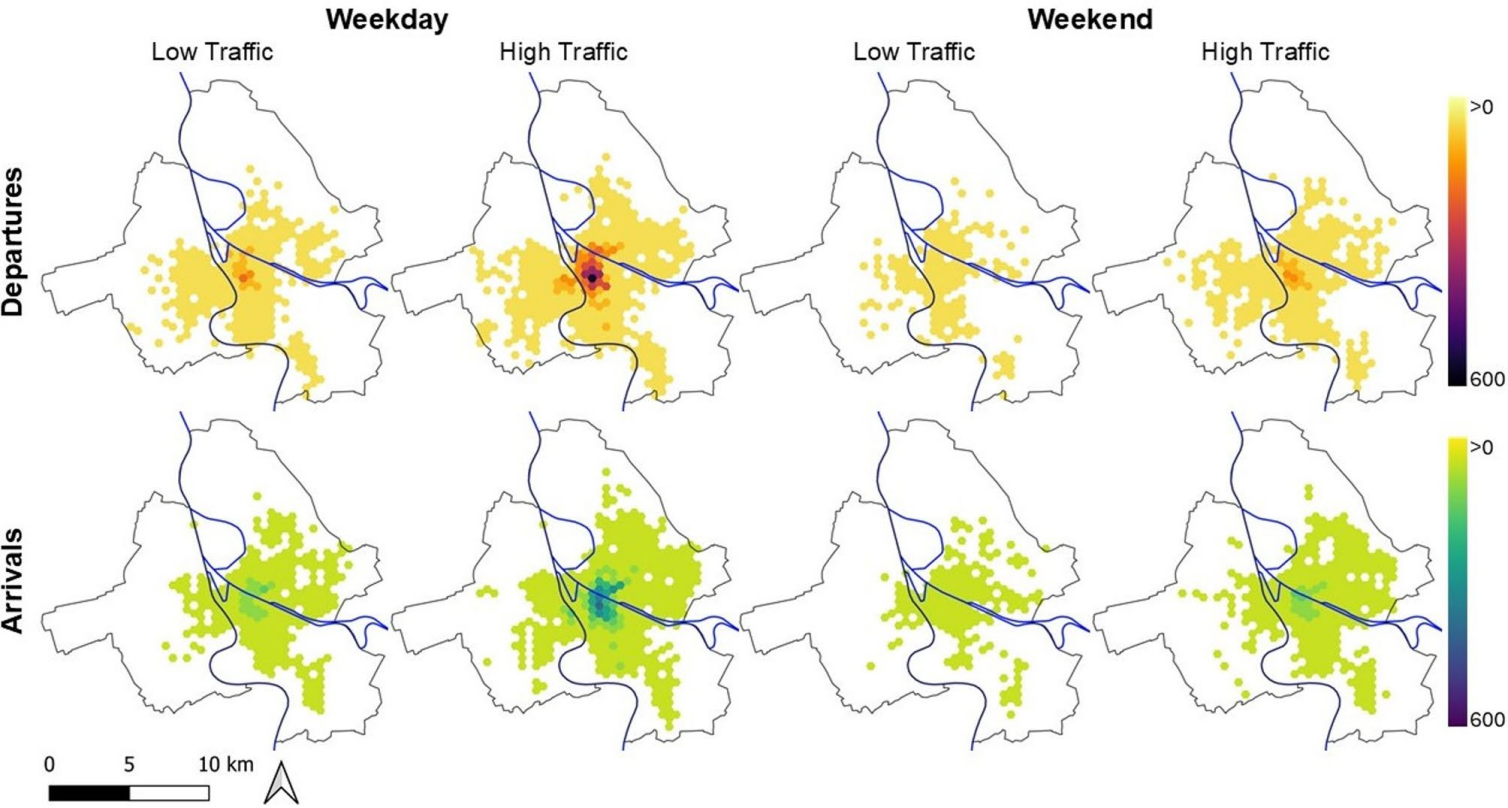
Spatial distribution of accumulated departures and arrivals by time period.

Figure 1 depicts the number of e-scooter departures and arrivals per time period, aggregated to a hexagonal grid with horizontal and vertical spacing of 500 m. It is visible that the hotspots of both departures and arrivals lie in the city centre of Mannheim, between the rivers Rhein and Neckar. In the case of Weekday High Traffic period, a second but smaller centre of demand lies in the city centre of Ludwigshafen. The locations of the trip arrivals are less concentrated than of the trip departures with the maximum number of e-scooter departures in a grid cell reaching up to 550 during Weekday High Traffic but arrivals per grid cell accumulating to only 372 in the same time period.

### Route choice modelling

To examine context-specific routing decisions, we analyse temporal variations in routing models across each of the four time periods. Per time period, four different model types are estimated (Table 1): Model 1 takes into account only attributes concerning the alternatives’ lengths and their bicycle infrastructure separation. Model 2 includes angles and turns and Model 3 relies solely on spatial configuration parameters. Model 4 comprises a combination of the attributes of Models 1, 2, and 3. The parameter estimation results for these models are presented in Table 4 and discussed below.

**Table 4:** Model parameter estimates (*p < 0.1, **p < 0.01, ***p < 0.001).

Parameter	Weekday low traffic				Weekday high traffic			
	Model 1	Model 2	Model 3	Model 4	Model 1	Model 2	Model 3	Model 4
Length [m]	− 0.0027***			− 0.0020***	− 0.0022***			− 0.0016***
ln* (PS*_*j*_*)*	2.33***			2.2900***	2.3100***			2.2600***
***Infrastructure***								
Contra road [m]					− 0.0016***			− 0.0014***
Contra road allowed [m]	− 0.00001			− 0.0002	− 0.0001			− 0.0001
Shared lane [m]	0.0019**			0.0014	0.0010			0.0004
Cycle lane [m]	0.0026***			0.0027***	0.0011***			0.0011**
Pedestrian space [m]	0.0001			0.0004**	− 0.0006***			− 0.0004***
Cycle track [m]	0.0022***			0.0021***	0.0015***			0.0013***
Contra cycle track [m]	0.0009***			0.0008**				
***Turns and angles***								
Turns right sharp [m^−1^]		− 0.5750***		0.0235***		− 182***		− 6.7300
Turns right right [m^−1^]						− 442***		− 146***
Turns left sharp [m^−1^]						− 179***		− 13.800
Turns left right [m^−1^]						− 438***		− 136***
***Spatial configuration***								
NACH 500 m min [–]							4.24***	1.47***
NACH 500 m mean [–]								
NACH 500 m max [–]			− 8.700***	− 4.9900***			− 8.61***	− 3.97***
TD maximum [–]			− 0.000005***	− 0.000003***				
Final log likelihood	− 4876.347	− 6682.339	− 7278.475	− 4485.643	− 11,201.83	− 18,844.25	− 16,388.04	− 9717.55

Parameter	Weekend low traffic				Weekend high traffic			
	Model 1	Model 2	Model 3	Model 4	Model 1	Model 2	Model 3	Model 4
Length [m]	− 0.0028***			− 0.0017***	− 0.0024***			− 0.0016***
ln* (PS*_*j*_*)*	2.2600***			2.2300***	2.4100***			2.4200***
***Infrastructure***								
Contra road [m]								
Contra road allowed [m]	− 0.0003			− 0.0007				
Shared lane [m]	0.0040*			0.0022	0.0040***			0.0033***
Cycle lane [m]	0.0028***			0.0023***	0.0018***			0.0017***
Pedestrian space [m]					− 0.0002			0.0003*
Cycle track [m]	0.0022***			0.0026***	0.0018***			0.0016***
Contra cycle track [m]								
***Turns and angles***								
Turns right sharp [m^−1^]		− 296***		84.6***		− 189***		53.2***
Turns right right [m^−1^]								
Turns left sharp [m^−1^]								
Turns left right [m^−1^]								
***Spatial configuration***								
NACH 500 m min [–]			1.49***	0.534			1.49***	0.548**
NACH 500 m mean [–]			2.53*	4.22**			1.61**	2.36***
NACH 500 m max [–]			− 10.7***	− 7.9***			9.1***	− 6.54***
TD maximum [–]			− 0.000003***	− 0.000002***			− 0.000004***	− 0.000002***
** Final log likelihood**	− 982.6342	− 1861.813	− 1432.606	− 882.6045	− 4914.076	− 8348.334	− 7153.219	− 4440.326

#### Infrastructure type and route length

Across Model 1 and 4, the consistently negative length parameters confirm behavioural validity, as longer distances reduce the likelihood of an alternative route being chosen. Most infrastructure attributes, particularly the separation between bicycle infrastructure and motorised traffic, increase the odds of an alternative being selected. In contrast, travelling in the contra-flow direction of a one-way road—whether legally permitted or not—is consistently associated with lower choice probabilities. Notably, only 36% of all observed trips did not use a one-way road illegally in a contraflow direction.

The influence of pedestrian space on the choice probability varies across the models, indicating time-dependent preferences among e-scooter riders. During weekday low-traffic times, pedestrian spaces positively affect choice probability, whereas during weekday high-traffic hours, they have a negative impact. The weekend models do not indicate a clear preference for pedestrian spaces, with high covariances leading to the removal of the attribute from the low traffic models, and Model 1 and Model 4 showing opposite effects for the high traffic times.

#### Turns

Regarding the impact of turns, Model 2, across time periods, consistently indicates that a higher number of sharp right turns reduces the likelihood of a route being chosen. However, in Model 4, the direction of this effect reverses, except during weekday high-traffic hours, where sharp and right-angled turns, both right and left, negatively impact choice probability. This shows that the influence of sharp right turns on e-scooter routing behaviour is context-dependent and time-sensitive, highlighting the importance of incorporating temporal variations in route choice models.

#### Spatial configuration

Model 3 focuses exclusively on spatial configuration parameters. “Normalised Angular Choice” (*NACH*), “Normalised Angular Integration” (*NAIN*), and “Total Depth” (*TD*) at varying radiuses, ranging from 500 m via 1 km, 5 km, 10 km, to the complete network, were tested for their potential impact on route choice as past research has found they impact aggregated traffic flows of different modes^19,66–68^. Due to covariances, a maximum of four significant variables were retained in the final models. Significant parameters are linked to the values of *NACH 500 m*, a measure for the through-movement potential of a network segment at a local level^66^, and *TD*, a metric describing the closeness of a given street segment to the other street segments. As these metrics constitute qualities of the network segments, they are assigned to the alternative routes by examining their minimum, mean, and maximum values along the alternative route.

The maximum value of *TD* is consistently associated with a lower choice probability, suggesting that e-scooter riders avoid areas that are not easily accessible for to-movement during weekends and weekday low-traffic periods. Similarly, the maximum value of *NACH 500 m* along the alternative route is consistently associated with a lower choice probability, apart from weekend high traffic times. In contrast, a higher minimum *NACH 500 m* value generally increases the choice probability, except during weekday low-traffic periods. Weekend models further reveal that a higher mean *NACH 500 m* value along the alternative route increases its choice probability. While the spatial configuration parameters can be interpreted as representing local through-movement or regional accessibility for to-movement potential^69^, they are abstract metrics and unlikely to be consciously considered by riders. Despite this, our model results highlight the clear influence of spatial configuration on routing behaviour. This does not only support existing research on the broader impact of urban structure on accumulated movement flows^70–72^ but also emphasises its influence on individuals’ routing decisions, a fact that should be taken into urban planning considerations.

#### Parameter combination

Model 4 integrates all parameters from Models 1, 2, and 3. As expected, the aggregate model fit improves, as indicated by higher final log-likelihood values, which measure how well the model predicts observed choices. Further, likelihood ratio tests are used to assess whether the more complex Model 4 significantly outperforms the simpler Models 1, 2, and 3, with the null hypothesis of ‘no improvement in goodness-of-fit’ being rejected at a significance level of 0.001. This demonstrates that combining infrastructure, turn, and spatial configuration attributes substantially enhances the predictive accuracy of the route choice model, highlighting the complexity of interactions between these parameters.

#### Value-of-distance indicators

To enhance the comparability of the effect sizes across different models with parameter estimates of varying magnitudes, marginal rates of substitution can be calculated. They provide insights into the trade-off between a baseline attribute and other attributes of the alternative within the utility function which, within a linear model, is achieved by dividing a parameter estimate with the baseline attribute’s parameter^73^. Table 5 provides an overview of the value-of-distance (VoD) indicators, marginal rates of substitution with the route’s alternative as the baseline attribute, which is typically chosen due to its straightforward interpretability, for the estimated models. This means the VoD indicator of route attribute *k* is calculated as *β*_*k*_ /* β*_*length*_. A negative VoD indicator suggests a reduction of the perceived distance of the alternative by the calculated fraction, and a positive VoD indicator the opposite. As Model 2 and Model 3 do not contain a length parameter, no value-of-distance indicators are calculated for them.

**Table 5:** Value-of-distance indicators.

Parameter	Weekday				Weekend			
	Low traffic		High traffic		Low traffic		High traffic	
	Model 1	Model 4	Model 1	Model 4	Model 1	Model 4	Model 1	Model 4
***Infrastructure***								
Contra road [m]			0.71	0.91				
Contra road allowed [m]	0.01	0.09	0.03	0.08	0.12	0.42		
Shared lane [m]	− 0.69	− 0.71	− 0.45	− 0.27	− 1.41	− 1.26	− 1.69	− 2.08
Cycle lane [m]	− 0.98	− 1.34	− 0.51	− 0.68	− 1.01	− 1.33	− 0.77	− 1.08
Pedestrian space [m]	− 0.02	− 0.22	0.27	0.28			0.09	− 0.20
Cycle track [m]	− 0.84	− 1.04	− 0.70	− 0.82	− 0.78	− 1.26	− 0.75	− 1.03
Contra cycle track [m]	− 0.33	− 0.38						
***Turns and angles***								
Turns right sharp [m^−1^]		− 11.87		4259.49		− 49,473.68		− 33,670.89
Turns right right [m^−1^]				92,405.06				
Turns left sharp [m^−1^]				8734.18				
Turns left right [m^−1^]				86,075.95				
***Spatial configuration***								
NACH 500 m minimum [–]				− 930.38		− 312.28		− 346.84
NACH 500 m mean [–]						− 2467.84		− 1493.67
NACH 500 m maximum [–]		2520.20		2512.66		4619.88		4139.24
TD maximum [–]		0.00				0.00		0.00

Table 5 shows that dedicated cycling infrastructure—which can also be used by e-scooters—such as dedicated cycle lanes (i.e. *β*_*Cycle lane*_ / *β*_*length*_) and cycle tracks (i.e. *β*_*Cycle track*_ / *β*_*length*_) consistently reduce the perceived distance by at least 51% (in the case of Weekday High Traffic Model 1), and at most by 134% (for the Weekday Low Traffic Model 4). The latter value suggests an overcompensation of the negative impact of route length on the alternative’s utility by providing cycling infrastructure. Riding an e-scooter in the contraflow direction of a road consistently increases the perceived distance with the effect being smaller when this practice is permitted. The effect size of turns is remarkable, even when it is considered that the seemingly four-digit numbers are reduced if the attribute unit is changed to, for instance, km^−1^ instead of m^−1^.

The similarity in effect size of the *NACH 500 m maximum* parameters for the weekday and weekend models respectively is noticeable, with the VoD indicator being more than 1.5 times higher on weekends than on weekdays. This indicates that local attractors of through-movement, especially for pedestrians, have a stronger negative effect on a route’s utility on the weekend than on weekdays which could be linked to increased pedestrian activities in local high streets during weekends which are avoided by e-scooter users. Similarly, across all models, the effect of the *TD maximum* parameter is small and not displayable with two decimal places.

#### Sensitivity analysis

**Fig. 2:**
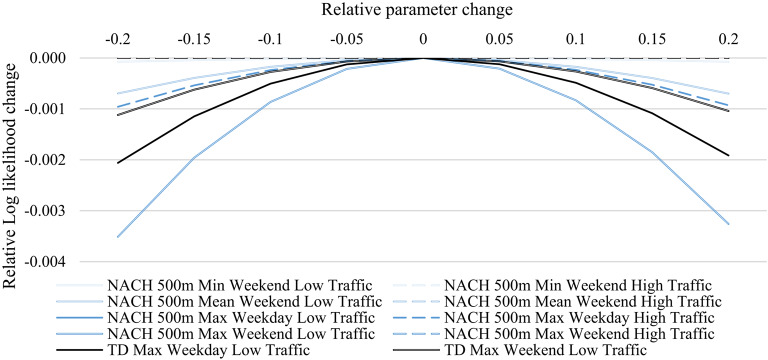
Sensitivity analysis of spatial configuration parameters.

To evaluate the stability of the modelling results in light of variable parameter estimates of the spatial configuration attributes introduced through this study, a sensitivity analysis is conducted. Specifically, we test the change of the model fit in light of a change in the spatial configuration parameters which we vary in 5% steps between 20% below and 20% above the parameter estimate. Figure 2 depicts the results of this analysis with the x-axis comprising the relative change in the spatial configuration parameters and the y-axis comprising the corresponding change in the model fit assessed through its log likelihood relative to the model with the estimated parameter.

Since a maximum likelihood estimator was used, it is visible that all models have their maximal model fit with the estimated parameters. While all parameters associated with a model describing a High Traffic period, i.e. Weekday High Traffic or Weekend Low Traffic, result in a relative change in the log likelihood of − 0.096% when the parameter is varied by 20%, the *TD maximum* parameter shows a relative variability of up to − 0.206% for the High Traffic periods, and *NACH 500 m maximum* of up to -0.196% for the case of Weekend Low Traffic. These results indicate that the choice of the spatial configuration parameters has little bearing over the models’ fits and that potential mis-estimations do not undermine the model quality.

## Discussion

This study reveals that e-scooter routing behaviour is shaped by a complex interplay of factors, including dedicated cycling infrastructure, route directness, spatial configuration, and temporal dynamics. Contrary to simplified assumptions in existing literature^74^, riders do not simply follow the shortest path. Instead, they show a clear preference for routes with a higher proportion of dedicated cycling infrastructure, such as segregated bicycle lanes and tracks, while avoiding routes with high maximum local *NACH 500 m* and maximum *TD*, reflecting a tendency to avoid places that are both highly accessible for through-movement on a local and comparatively secluded on a regional level.

The results contribute to the existing body of space syntax theory-driven knowledge by integrating spatial configuration parameters into discrete route choice models, whereas traditional space syntax studies focus on accumulated flows. Specifically, we demonstrate for the first time, based on large-scale tracking data on a city level from e-scooters, that the spatial configuration parameters *NACH 500 m* and *TD maximum* can substantially improve the models’ goodness-of-fit. Our study’s findings show that e-scooter routing behaviour occupies an intermediate position between pedestrian and vehicular flows as angular choice measures have been strongly correlated with vehicle flows^21^, whereas *NACH* measures typically have limited influence on pedestrian traffic^68^. This finding substantially broadens the application of space syntax to emerging transport modes, and call for integrating spatial configuration into route choice models for all modes of significance.

The perceived value of cycling infrastructure is particularly notable, with cycle lanes and tracks reducing travel distance by at least 51%. This aligns with previous research showing that both current and potential e-scooter users consider dedicated infrastructure as a means to avoid potentially dangerous interactions with motorised traffic^64,75,76^. Apart from weekday high traffic times, the positive impact of cycling infrastructure even outweighs the negative impact of increased route length, further underscoring its importance. These findings are also consistent with cycling-focused studies^51,59,62^ and emphasise the value of investment in such infrastructure for planning sustainable cities.

Temporal variations indicate that e-scooter riders utilise pedestrian spaces differently depending on traffic conditions. Pedestrian areas are preferred during weekday low-traffic periods, likely as a way to avoid congested roadways, but are avoided during weekday as well as weekend high-traffic times. This suggests that pedestrian spaces serve as a deviation when carriageways are perceived as too busy during car traffic peak hours but are not a preferred option otherwise. Also, a lower number of e-scooters in use could lead to a rider’s reduced perception of safety when using a carriageway as they anticipate a reverse “safety in numbers” effect^77^, leading to e-scooter riders avoiding potentially conflict-prone road spaces.

Interestingly, despite e-scooter use in pedestrian areas being illegal in the study region^78^, the data reveal an average of 209 m per trip in such spaces, with fewer than 30% of trips avoiding pedestrian areas entirely. It needs to be considered, however, that the dataset does not distinguish between ridden and pushed e-scooters, which may influence these findings, as users can jump off and on their vehicles^11^. Overall, these results suggest that riders often perceive roadways as unsafe or congested, reinforcing the need for policymakers to expand micro-mobility infrastructure on high-demand roads and reduce reliance on pedestrian spaces. Integrated planning that incorporates both pedestrian and micro-mobility needs is essential to addressing conflicts and enhancing the overall functionality of urban spaces.

Future research should explore the generalisability of these findings by conducting comparative studies in different geographic and regulatory contexts. This is particularly relevant as the data used to estimate the models is likely to be affected by the events surrounding Covid-19. While most legal restrictions limiting the movement and social interactions were lifted during the study period, potentially temporal behavioural changes could have affected people’s trip purposes, times and frequencies, people’s mode choices and the composition of the e-scooter ridership, the business of streets, etc.

To improve the accuracy of the route choice models, future work should also incorporate additional route attributes and user characteristics which could not be incorporated into the presented models due to a shortage of available data. For example, factors influencing route attractiveness outside of dedicated cycling infrastructure and intuitiveness, such as the volume of vehicular traffic captured through vehicular counts or derived from traffic models, the proximity of green and blue infrastructure based on geospatial data, perceptions of safety and pleasantness identified through field surveys, as well as the state of the infrastructure based on datasets typically held by local governments could be tested for their relevance to e-scooter route choice. In addition, it can be hypothesised that, although the physical effort required to ride an e-scooter uphill is less than for a bicycle, slope could still impact battery charging levels and rider comfort and therefore, its relevance for e-scooters’ routing should be assessed. Furthermore, incorporating user characteristics, such as experience levels, demographics, or familiarity with the area, could further enhance the predictive accuracy of route choice models. Additionally, understanding the behaviour of riders of private e-scooters, who may have different preferences than shared e-scooter users, is crucial for developing inclusive transport policies and infrastructure strategies.

The results of this study provide actionable insights for policymakers, planners, and businesses. Transport planners can use these findings to incorporate e-scooters into existing transport models, enabling the assessment of potential impacts from changes in land use, employment changes, and infrastructure development. In particular, this can help local authorities to justify investments in cycling infrastructure as part of a unified micro-mobility network, including converting one-way cycle tracks into bi-directional lanes to accommodate growing demand.

For shared e-scooter operators, these findings can inform fleet management strategies. Recognising user preferences for specific routes due to infrastructure, safety, or convenience can help anticipate demand hotspots and optimise fleet allocation and charging strategies. Frequently chosen routes that deviate from the shortest paths can guide the placement of charging stations or swapping hubs, reducing inefficiencies. Operators can also implement dynamic pricing strategies that reflect common trip patterns, incentivising users to take specific routes that balance system utilisation or avoid congestion. These improvements collectively enhance the sustainability and efficiency of e-scooter systems, benefiting urban mobility as a whole.

## Methods

### Data

#### Network

Factors typically considered in route choice models range from costs and time or distance measures to attributes of the built environment and quality of the available infrastructure to geometric aspects of the chosen route. As for cars, bicycle route choice models typically include attributes linked to the infrastructure of the choice set in question. This comprises the level of separation of bicycle infrastructure from other forms of transport, particularly cars^52,60–63^, one-way restrictions^63^, type of intersection control^51,63^, slope^51,62^, and traffic levels^51,63^.

Information about infrastructure useable by e-scooters has been extracted from OpenStreetMap (OSM)^79^, including information about separate bicycle infrastructure that can be used by e-scooters, and handled with QGIS to accommodate the route choice modelling process. Roads that are not useable by e-scooter users (e.g., motorways and dual carriageways) are omitted from the infrastructure dataset.

**Fig. 3:**
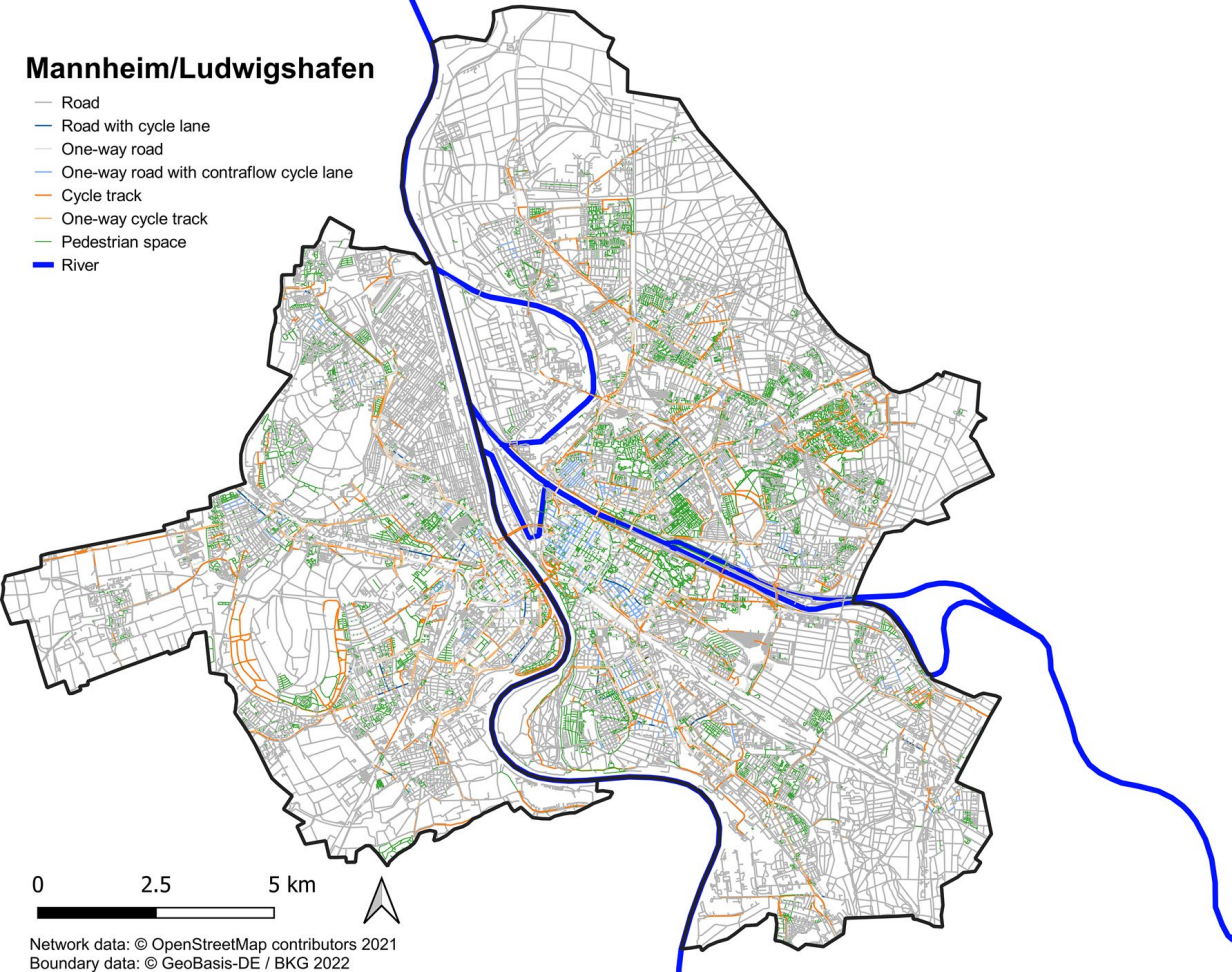
Network classification in the Mannheim/Ludwigshafen metropolitan area.

Building on previous research indicating that e-scooter users prefer bicycle infrastructure, the OSM network is classified into four categories: (i) roads with no separate bicycle infrastructure, (ii) roads with bicycle lanes, (iii) cycle tracks, and (iv) areas designated for pedestrian use. Furthermore, one-way restrictions, including exemptions for bicycle users, are identified from the OSM dataset. Figure 3 and Table 6 depict the distribution of bicycle infrastructure in Mannheim/Ludwigshafen, Germany.

**Table 6:** Network classes in the Mannheim/Ludwigshafen metropolitan area.

Infrastructure	Length [km]
Road	3580.0
… with bicycle lane	36.5
… one-way	497.1
… with bicycles allowed contraflow	45.7
Bicycle track	404.3
… one-way	105.0
Pedestrian space	716.6
Total	4700.9

Spatial configuration can be operationalised using the methods provided by space syntax theory that builds on graph theory to analyse the position of one spatial entity relative to all or parts of the totality of all spatial entities. In research, these spatial entities have been axial lines, describing longest lines of sight^20,80^, segment lines, capturing the connections between intersections of axial lines^81^, or road centre lines^21^. As this research is concerned with capturing the differences between different infrastructure qualities—something that cannot be integrated into the axial lines approach—and needs to consider the length of routes—that cannot be captured with either the axial lines or the segment lines approach—the latter approach is chosen.

Researchers in space syntax theory have developed multiple key parameters to analyse a place’s spatial configuration. This study follows the works of Ståhle et al.^82^ and van Nes and Yamu^69^ that have been integrated into the Place Syntax Tool plugin for QGIS, which has been used to calculate the space syntax parameters. They are based on the construction of a graph from street network data with, for the purpose of this study, a street segment being interpreted as a node of the graph and intersections creating links between them. In that context, the “Total Depth” of segment *u*, *TD(u)*, is defined as the sum of shortest walking distances *d*_*walking*_*(u,v)* from *u* to every other segment *v* in the network:

1 $$TD\left( u \right) = \mathop \sum \limits_{u \ne v}^{N} d_{walking} \left( {u, v} \right),$$

with *N* as the total number of segments in the network.

In addition to walking distance, path lengths can be measured as angular distance. For example, the space syntax research community has developed the attributes of “Angular Integration” *AI,* “Normalised Angular Integration” *NAIN*, and “Normalised Angular Change” *NACH* of a street segment *u*. For completeness, these values are defined in Eqs. (2–4) below^82^, although the former two attributes were not retained for any of the final models presented in this article:

2 $$AI\left(u\right)= \frac{N-1}{1+{\sum }_{u\ne v}{d}_{angular}\left(u,v\right)}$$

3 $$NAIN\left(u\right)=\frac{{N}^{1.2}}{1+{\sum }_{u\ne v}{d}_{angular}\left(u,v\right)}$$

4 $$NACH\left(u\right)= \frac{\text{log}({CH}_{angular}(u) + 1)}{\text{log}(2+ {\sum }_{u\ne v}{d}_{angular}\left(u,v\right))}$$

The angular distance between nodes *u* and *v*, *d*_*angular*_*(u,v)*, is measured as a function of angular change (Eq. 5):

5 $${d}_{angular}\left(u,v\right)= \underset{P\in {\mathcal{P}}_{u,v}}{\text{min}}{\sum }_{i=1}^{{N}_{P}-1}w({\gamma }_{i, i+1})$$

with ${\mathcal{P}}_{u,v}$ as the set of paths between *u* and *v*, and *γ*_*i,i*+*1*_ as the angle between two subsequent segments *i* and *i* + *1* within a path *P* with *N*_*P*_ segments, measured in radian.

The angular weight is defined in Eq. (6) and ensures that straight lines which are split into segments at intersections and therefore do not constitute a turn for the traveller, have a weight of 0 and right angles a weight of 1 as demanded by Hillier and Iida^83^:

6 $$w\left({\gamma }_{i, i+1}\right)= 2-\frac{2}{\pi }({\gamma }_{i, i+1} mod \pi )$$

“Angular Choice” *CH*_*angular*_ of segment *u*, or, in other words, the betweenness centrality^69^ of node *u* in a graph with link weights depending on the angle between the segments is the number of shortest angular paths between nodes *s* and *t* in the network (Eq. 7):

7 $${CH}_{angular}\left(u\right)= {\sum }_{f}^{N-1}{\sum }_{g}^{N-1}\frac{{\sum }_{t}^{{|\mathcal{P}*}_{f,g}|}{\delta }_{t}}{{|\mathcal{P}*}_{f,g}|}$$

with $u\ne f\ne g$ denoting segments in the network, ${\mathcal{P}*}_{f,g}$ the set of shortest angular paths between *f* and *g*, *N* the number of segments in the network, and ${\delta }_{t}$ = 1 if segment *u* is included in the *t*-th path and ${\delta }_{t}$ = 0 if not. Typically, ${\mathcal{P}*}_{f,g}$ would contain only one element, meaning ${|\mathcal{P}*}_{f,g}|=1$, but in rare occasions, multiple shortest paths of similar lengths can be found whose weights are then evenly distributed across the network.

These measures, if applied to a network as a whole, are called *global* and are sensitive to the boundary of the network^84^. This is mitigated if the *local* measures are calculated, i.e. for a subset of the network which is defined by a radius *r* around segment *u*.

#### Tracking data

Tracking data from 31,154 trips made with shared e-scooters during the period 1st June to 31st August 2021 in the twin city of Mannheim/Ludwigshafen in the states of Baden-Württemberg and Rheinland-Pfalz in the South-West of Germany have been provided by the micro-mobility company Lime. In addition to the trips’ start and end times, GPS coordinates have been recorded at intervals between 1 and 120 s. Obvious errors, represented by empty records and trips with a distance of 0, as well as unlikely trips, i.e., trips with an average velocity of 0.5 m/s or greater than 6 m/s—which represent walking speed^85^ and the legal speed limit^78^, respectively—are removed from the database.

The GPS tracks have been matched to the road network using a Hidden-Markov-Model (HMM)-based algorithm^86^. After additional filtering to remove map-matched trips with too high a discrepancy from the original track in terms of distance, speed, and detours, 14,029 trips’ records are used for the model estimation process.

To identify potential differences in routing between times of low and high e-scooter traffic, we classify every of the 168 h of a week as either being a low or a high traffic time. For this, the average number of trips over the course of the observation period is calculated for each of these 168 h. An hour is classified as “Low traffic” if its average number of trips is below the average of every hour and as “High traffic” if it is equal to or above that (Eq. 8):

8 $$h\in \left\{\begin{array}{c}\textit{Low traffic}, \text{if } {n}_{ave,h}<{n}_{ave, total}\\ \textit{High traffic}, \text{else}\end{array}\right.$$

with *h* as an hour of the week, *Low traffic* and *High traffic* being sets of hours of the week, *n*_*ave,h*_ as the average number of trips during a week’s *h*’s hour and *n*_*ave,total*_ as the average number of trips over every hour in the dataset.

### Route choice model

Route choice modelling is commonly based on random utility models, which assume that individuals aim to maximise the benefit derived from their choices. This benefit is typically expressed as the utility of each alternative, depending on the attributes of the alternatives and the characteristics of the decision-maker. However, due to the unavailability of data on the trip makers’ characteristics for this research, these characteristics cannot be considered when analysing the alternatives’ utility in this study.

#### Choice set generation

In theory, the number of potential routes between two points in space is unlimited. Therefore, identifying the set of choices available to and considered by the individual is a critical focus in revealed preference studies, especially in route choice modelling. For this study, the Metropolis–Hastings algorithm^87^ is applied to generate a set of alternatives. Starting from an initial route connecting an origin node *a* with a destination node *b* which typically is the shortest path between the two, the algorithm creates a representative sample of alternative routes available between *a* and *b* by adding nodes to and consequently deleting nodes from that path. The observed routes are by default added to the choice set.

On average, 25.7 alternatives are created per trip with a minimum of two and a maximum of 157 alternatives. For half of the observed trips, 22 or fewer alternatives have been created. The alternatives’ attributes are calculated based on the attributes of the utilised network segments. Table 7 provides an overview of the attributes of both the observed routes and their alternatives.

**Table 7:** Attribute statistics for observed and alternative routes.

	Observed routes				Alternative routes			
	Min	Max	Average	Std	Min	Max	Average	Std
Length [m]	8.50	16,243.60	1584.83	1364.73	8.50	42,550.14	2149.96	1964.31
***Infrastructure***								
Road [m]	0.00	8495.36	626.98	700.40	0.00	24,096.02	894.78	990.97
Contra one-way road [m]	0.00	3073.14	157.82	270.87	0.00	8987.05	357.73	438.29
Contra one-way road allowed [m]	0.00	1333.12	52.32	132.69	0.00	2521.56	76.15	155.70
Cycle lane [m]	0.00	2608.99	124.56	234.72	0.00	3591.45	103.77	178.39
Pedestrian space [m]	0.00	4892.55	209.04	299.11	0.00	5971.32	390.43	436.86
Cycle track [m]	0.00	6054.48	371.40	621.57	0.00	8048.46	278.05	457.83
Contra cycle track [m]	0.00	2083.13	36.50	147.53	0.00	2927.85	45.09	125.82
Shared lane [m]	0.00	451.71	6.22	39.47	0.00	455.82	3.95	28.14
***Turns and angles***								
Turns right sharp [m^−1^]	0	0.1177	0.0017	0.0032	0	0.1177	0.0022	0.0019
Turns right right [m^−1^]	0	0.1065	0.0014	0.0027	0	0.1065	0.0023	0.0020
Turns left right [m^−1^]	0	0.1065	0.0014	0.0026	0	0.1065	0.0023	0.0019
Turns right sharp [m^−1^]	0	0.1177	0.0017	0.0032	0	0.1177	0.0022	0.0019
***Spatial Configuration***								
NACH 500 m minimum [–]	0.00	1.53	0.73	0.28	0.00	1.53	0.57	0.26
NACH 500 m mean [–]	0.36	1.53	1.11	0.11	0.36	1.53	1.08	0.08
NACH 500 m maximum [–]	0.69	1.53	1.33	0.15	0.51	1.54	1.39	0.12
TD maximum [–]	5,347,097.3	10,620,805.0	6,587,742.7	768,244.4	5,347,097.3	11,474,090.0	6,804,808.9	785,326.6

Observed routes, on average, are shorter than the alternatives. For both the observed and the alternative routes, the proportion spent on road space without dedicated bicycle or pedestrian infrastructure, including legally or illegally riding in the contra sense of a one-way road, accounts for the largest part of an average trip. At intersections, the angle of a turn is measured, whereby a continuation as a straight line is equivalent to 180˚ and a U-turn is equivalent to 0. As expected, the smallest angles in both observed and alternative roads is equal to 0 whereas the largest angles are 180˚ in both cases. On average, the total turning angle along a route is less than half as big as in the generated alternatives. Furthermore, the observed routes, on average, involve fewer turns (i.e. changes of direction) than the generated alternatives. This is also true for sharp and right-angled turns, regardless of whether they are directed towards the right or left. Regarding the spatial configuration parameters, “Normalised Angular Change” (*NACH*), “Angular Integration” (*AI*), and “Total Depth” (*TD*) have been calculated for the network. These network parameters are translated into route attributes by calculating their minimum, maximum, and mean values. This approach is chosen as it allows to identify whether e-scooter riders avoid or prefer infrastructure of high or low centrality.

#### Modelling framework

The systematic utility of alternative *j V*_j_ is captured through a linear combination of the alternative’s attributes *X*_*jk*_ and parameters *β*_*jk*_ (Eq. 9)

9 $${V}_{j}= {\sum }_{k}{\beta }_{jk}\cdot {X}_{jk}+ {\beta }_{j}^{PS}\cdot {\text{ln}(PS}_{j})$$

with *k* as an attribute’s index, whereby *PS*_*j*_ describes the alternative’s Path Size which accounts for the overlap between alternative routes in within the choice set and is formulated as depicted in (Eq. 10) ^88^:

10 $${PS}_{j}= \sum_{i\in {\Gamma }_{j}}\frac{{l}_{i}}{{L}_{j}}\cdot \frac{1}{\sum_{j\epsilon C}{\delta }_{ij}}$$

with ${\Gamma }_{j}$ as the set of all segments in alternative route *j*, *l*_*i*_ as the length of segment *i*, *L*_*j*_ as the total length of route *j*, *C* as the route choice set, and *δ*_*ij*_ as 1 if link *i* is included in route *j* and 0 if not.

The choice probability *P*_*j*_ of alternative *j* is given through Eq. (11):

11 $${P}_{j}=\frac{\text{exp}({V}_{j})}{\sum_{i}\text{exp}({V}_{i})}$$

The python Biogeme package^89^ was used for model estimation.

## Data Availability

The data supporting the findings of this study are available from Lime Electric Ireland Limited but are not publicly accessible due to licensing restrictions. Access to the data may be granted upon request to Dr Haitao He, subject to approval from Lime Electric Ireland Limited.
